# Commentary: How to Make the Ghosts in my Bedroom Disappear? Focused-Attention Meditation Combined with Muscle Relaxation (MR Therapy)—A Direct Treatment Intervention for Sleep Paralysis

**DOI:** 10.3389/fpsyg.2017.00506

**Published:** 2017-04-03

**Authors:** Brian A. Sharpless, Karl Doghramji

**Affiliations:** ^1^Clinical Psychology Program, American School of Professional Psychology, Argosy University, Northern VirginiaArlington, VA, USA; ^2^Jefferson Sleep Disorders Center, Thomas Jefferson UniversityPhiladelphia, PA, USA

**Keywords:** sleep paralysis, isolated sleep paralysis, cognitive behavior therapy, parasomnia, sleep-wake disorders

We read with interest Dr. Jalal's recent publication on another potential treatment option for recurrent isolated sleep paralysis: *Meditation Combined with Muscle Relaxation* (MR Therapy). The author is to be commended for adding to the limited literature on ameliorating problematic cases of isolated sleep paralysis (ISP). As recently reviewed (Sharpless, 2016), there are few options available, pharmacological or otherwise, for the chronic and severe cases of ISP, and none of these yet possess compelling evidence in favor of their efficacy.

We appreciate the citation of our work in his article. However, we disagree with the author's claim that MR Therapy is the “first direct treatment” for ISP (Jalal, 2016, p. 1). This comment is puzzling given that our 2015 book (i.e., Sharpless and Doghramji, 2015) was cited in this same manuscript. Contained within is a manual titled *Cognitive Behavioral-Therapy for Isolated Sleep Paralysis* (CBT-ISP) and a corresponding adherence measure (pp. 237–272).

CBT-ISP is a brief, systematic approach that focuses upon helping patients both prevent and disrupt recurrent ISP episodes. In order to better clarify the nature of our own approach and to compare and contrast it to MR Therapy, we detail CBT-ISP in Table 1 below.

**Table 1:** **Cognitive behavior therapy for isolated sleep paralysis (CBT-ISP)**.

**PREPARATORY THERAPEUTIC ACTIVITIES**
Self-monitoring of ISP episodes via modified sleep diaries
Psychoeducation about the nature of ISP
Psychoeducation about the particular nature of ISP hallucinations (if present)
Presentation of the cognitive-behavioral model for ISP including the predictable “sequence” of ISP episodes (e.g., the cycle of symptoms, role of maladaptive appraisals, and increasing levels of activation)
In-session practice to dispute catastrophic thoughts associated with both paralysis and hallucinations (with instructions for homework)
Imaginary rehearsal of disruption techniques (e.g., focused attention to mobilize a finger/toe or making attempts to cough in order to foster the return of movement and dispel hallucinations) during session. This is done while the patient is in a supine position using a chaise/couch. Following imaginary rehearsal, the patient is provided with instructions for at home practice and *in vivo* application of these various disruption techniques *during* ISP episodes
**APPROACHES TO ISP EPISODE PREVENTION**
Use of ISP-specific sleep hygiene (e.g., ways to avoid sleep disruption, avoiding a supine sleeping position, limiting the use of certain substances prior to bed, removal of maladaptive avoidance behaviors)
Use of diaphragmatic breathing, progressive muscle relaxation, meditation, and/or mindfulness exercises to reduce overall anxiety levels throughout the day (i.e., not only during ISP episodes)
Relapse-prevention instructions are provided during the last therapy session using a “coping-based” approach
**APPROACHES TO ISP EPISODE DISRUPTION**
Applied diaphragmatic breathing, relaxation, mindfulness, or meditation during ISP episodes with attempts to “remain calm” (e.g., use of reassuring self-talk, use of distraction to focus attention away from hallucinations, reappraisal of episode/symptom meanings) in order to both disrupt and shorten ISP episodes
*In vivo* application of episode disruption techniques. If the initial attempt is ineffective, patients are instructed to flexibly apply secondary disruption techniques
Application of disruption techniques earlier and earlier in the predictable sequence of ISP episodes

*Adapted from Sharpless and Doghramji (2015). ISP, isolated sleep paralysis*.

As can be seen, there are areas of substantial overlap between CBI-ISP and MR Therapy. Both have strong emphases on various forms of relaxation, symptom reappraisals, shifts in attention away from episode content, and the practice of disruption techniques while patients are in a supine position. We should also note that both approaches are based upon panic disorder models.

However, there are some notable points of divergence. First, MR Therapy discourages attempts to move, whereas CBT-ISP actively encourages these attempts in order to directly disrupt episodes and shift attention away from potentially frightening symptoms (e.g., hallucinations). Our approach is based upon empirical work with sufferers who reported that these attempts were effective (Sharpless and Grom, 2016). Second, the author discourages attempts to control breathing whereas we view this as a potential source of relaxation to be used “in the moment.” Third, he encourages the use of prayer as a form of relaxation/meditation whereas we are more cautious about this, especially with patients who experience religious-themed hallucinations (e.g., demons, djinn). In general, CBT-ISP more strongly emphasizes psychoeducation, episode prevention, and consistent self-monitoring of ISP episodes (using modified sleep diaries) than MR Therapy, but both agree that directly disrupting ISP is important.

It is obviously preferable to have multiple treatment approaches available in a clinician's armamentarium as opposed to one. Efficacy rates for ISP approaches are not yet known, and it is very likely that both of these approaches will need to be modified in response to emerging data.

In closing, we believe that CBT-ISP (Sharpless and Doghramji, 2015) is the first direct treatment for ISP. However, it may be important to note that academic debates about temporal priority and differences in treatment foci are really moot points unless the phenomenon of recurrent ISP is more frequently assessed in research and clinical practice. This does not yet appear to be the case. At least two studies to date have shown that a minority of patients experience ISP to such a degree that it is associated with significant clinical consequences (Sharpless et al., 2010; Sharpless and Grom, 2016). Unfortunately, at the time of this writing there are not yet any empirically-supported treatment options available for sufferers.

## Author contributions

All authors listed have made substantial, direct, and intellectual contribution to the work, and approved it for publication.

### Conflict of interest statement

Both authors have received royalties from Oxford University Press for the publication of their book Sleep Paralysis: Historical, Psychological, and Medical Perspectives.
